# Dysregulated miRNA in a cancer-prone environment: A study of gastric non-neoplastic mucosa

**DOI:** 10.1038/s41598-020-63230-1

**Published:** 2020-04-20

**Authors:** Binnari Kim, Jiryeon Jang, You Jeong Heo, So Young Kang, Heejin Yoo, Insuk Sohn, Byung-Hoon Min, Kyoung-Mee Kim

**Affiliations:** 1 Department of Pathology and Translational Genomics, Samsung Medical Center, Sungkyunkwan University School of Medicine, Seoul, Republic of Korea; 2 Center of Companion Diagnostics, Samsung Medical Center, Seoul, Republic of Korea; 3 The Samsung Advanced Institute for Health Sciences & Technology (SAIHST), Samsung Medical Center, Sungkyunkwan University School of Medicine, Seoul, Korea; 4 Statistics and Data Center, Samsung Medical Center, Sungkyunkwan University School of Medicine, Seoul, Republic of Korea; 5 Department of Medicine, Samsung Medical Center, Sungkyunkwan University School of Medicine, Seoul, Republic of Korea

**Keywords:** Cancer, Biomarkers, Gastroenterology

## Abstract

Understanding cancer-prone environments is important to efficiently detect and prevent cancers. The associations between miRNA and cancer-prone environments are still largely unknown in gastric cancer (GC). Six miRNAs that are differentially expressed during gastric carcinogenesis were selected, and quantitative real-time PCR was performed in an independent training set (fresh non-tumor and tumor samples from 18 GC patients) and validation sets (set 1 with formalin-fixed paraffin-embedded non-tumor and tumor samples from 19 solitary GC and set 2 with 37 multiple GC patients). The results were compared with those of 37 gastric mucosa from 20 healthy volunteers. The expression levels of miR-26a, miR-375, and miR-1260 in gastric mucosa from healthy volunteers were statistically higher than that of non-tumorous gastric mucosa located 3 cm apart from the GC in the training set (miR-26a, *P* < 0.0001; miR-375, *P* = 0.0049; miR-1260, *P* = 0.0172), validation set 1 (miR-26a and miR-375, *P* < 0.0001; miR-1260, *P* = 0.0008), and validation set 2 (miR-26a, miR-375, and miR-1260, *P* < 0.0001). And a combination of miR-26a and miR-1260 showed the highest area under the curve value of 0.89. miRNAs are differentially expressed in non-neoplastic gastric mucosa and can be used as a biomarker to predict cancer-prone environments.

## Introduction

An understanding of premalignant lesions is very important to efficiently detect and prevent cancers. Recently, the concept of “Pre-Cancer Genome Atlas (PCGA)”, similar to The Cancer Genome Atlas (TCGA), has been developed to characterize the molecular alterations in premalignant lesions and “cancer-prone” environments, a non-neoplastic histopathology accompanied by genetic or epigenetic changes^1^. This PCGA would characterize the genetic alterations in premalignant lesions and corresponding changes in the microenvironment across multiple tumor types for early cancer detection and prevention^1^. Although studies of premalignant lesions have been carried out^2–4^, the associations of miRNA with cancer-prone environments are insufficient compared to TCGA data and are mainly studied in advanced stage cancers.

MicroRNAs (miRNA) are endogenous, non-coding single strand RNAs that inhibit translation or promote degradation of messenger RNAs with complementary sequences. miRNAs are differently expressed depending on cell types and tissues. miRNAs play a key role in the regulation of diverse biological processes at translational and post-transcriptional levels^5,6^. Many studies have revealed aberrant miRNA expression associated with diseases, especially cancers, and altered miRNA expression has been observed in various kinds of cancer^7–10^.

In gastric cancer (GC), many papers have reported miRNA dysregulation as a new biomarker^11–19^. In our previous work, we showed that a gradual increase or decrease of miRNA expression along with histologic progression from normal, low grade dysplasia, high grade dysplasia, and early gastric carcinoma; we also demonstrated that miRNA is involved in gastric carcinogenesis^2^. There have been a few studies on cancer-prone environments of GC^3,4,20,21^; however, these papers included small samples without test cohorts^4^, no clear definition of ‘cancer adjacent’^4^, no comparison between antrum and body^3^, and focused on methylation-associated silencing of miRNA instead of miRNA expression^20,21^.

To shed light on the role of miRNA in cancer-prone environments, six representative miRNAs were selected based on our previous work^2^ and quantitative real-time PCR was performed to compare miRNA expression in normal gastric mucosa of healthy young volunteers with that of non-tumor and tumor samples in patients with GC. In addition, RNA sequencing data was used to evaluate the differentially expressed target genes of those miRNA.

## Results

### Dysregulated miRNA expression within normal gastric mucosae from healthy volunteers and non-tumor mucosae from gastric cancer patients

In a training set, miR-26a expression in normal mucosa from healthy volunteers was significantly higher than that of non-tumorous gastric mucosa located 3 cm (*P* < 0.0001) and 1 cm (*P* < 0.0001) apart from the tumor. The miR-375 expression in normal mucosa from healthy volunteers was significantly higher than that of non-tumor mucosa at 3 cm (*P* = 0.0049) and 1 cm apart from the tumor (*P* = 0.0034). The miR-1260 expression in normal mucosa from healthy volunteers was significantly higher than that of non-tumor mucosa 3 cm (*P* = 0.0172) and 1 cm apart from the tumor (*P* < 0.0001) (Table 1).

**Table 1:** Differences of miRNA expression between gastric mucosae from healthy volunteers and non-tumor tissues which are 3 cm apart from tumors (A), and 1 cm apart from tumors (B) in a training set.

A						
Risk factors	miR-26a		miR-375		miR-1260	
	β coefficient	p-value	β coefficient	p-value	β coefficient	p-value
GC patients (3cm) vs healthy volunteers	−6.42 ± 0.94	**<0.0001**	−5.96 ± 2.12	**0.0049**	−5.93 ± 2.49	**0.0172**
M vs F	−0.31 ± 0.67	0.6455	0.66 ± 1.03	0.522	2.21 ± 1.63	0.1751
Age	0.06 ± 0.03	**0.037**	0.06 ± 0.06	0.2813	0.03 ± 0.06	0.5909
IM (3) vs IM (0-2)	−0.94 ± 0.92	0.3042	−0.02 ± 2.43	0.992	0.82 ± 3.44	0.8124
Atrophy (3) vs Atrophy (0-2)	1.41 ± 1.37	0.3002	0.59 ± 1.89	0.7549	−3.11 ± 2.23	0.1633
**B**						
**Risk factors**	**miR-26a**		**miR-375**		**miR-1260**	
	**β coefficient**	**p-value**	**β coefficient**	**p-value**	**β coefficient**	**p-value**
GC patients (1cm) vs healthy volunteers	−6.80 ± 0.87	**<0.0001**	−6.60 ± 2.25	**0.0034**	−8.54 ± 1.85	**<0.0001**
M vs F	0.11 ± 0.62	0.8549	0.54 ± 1.06	0.6092	1.81 ± 1.57	0.2495
Age	0.05 ± 0.03	0.0645	0.05 ± 0.06	0.3931	0.02 ± 0.06	0.6922
IM (3) vs IM (0-2)	−0.34 ± 0.41	0.411	3.51 ± 2.04	0.0852	4.03 ± 2.26	0.0744
Atrophy (3) vs Atrophy (0-2)	0.06 ± 0.42	0.8777	−3.95 ± 2.06	0.0554	−4.71 ± 2.45	0.0543

GC, gastric carcinoma; IM, intestinal metaplasia.

When comparing miRNA expression between non-tumor mucosa 3 cm away from the tumor and 1 cm away from the tumor, there was also a significant difference in all miRNAs (miR-26a, *P* = 0.0483; miR-375, *P* = 0.0304; and miR-1260, *P* = 0.0016) (Supplementary Fig. 1).

In validation set 1, miR-26a and miR-375 expression in the normal mucosa from healthy volunteers was significantly higher than that of non-tumor mucosa from patients with GC (*P* < 0.0001). The miR-1260 expression of normal mucosa from healthy volunteers was significantly higher than that of non-tumor mucosa from patients with solitary GC (*P* = 0.0008) (Table 2A). In validation set 2, miR-26a, miR-375, and miR-1260 expression in normal mucosa from healthy volunteers was significantly higher than that of non-tumor mucosa from patients with multiple GCs (*P* < 0.0001) (Table 2B).

**Table 2:** Differences of miRNA expression between gastric mucosae from healthy volunteers and non-tumor tissues from gastric carcinoma patients in validation sets.

A. Validation set 1						
Risk factors	miR-26a		miR-375		miR-1260	
	β coefficient	*P*-value	β coefficient	*P*-value	β coefficient	*P*-value
GC patients vs. healthy volunteers	−6.45 ± 1.24	**<0.0001**	−6.37 ± 1.55	**<0.0001**	−6.65 ± 1.99	**0.0008**
M vs. F	0.96 ± 0.72	0.1827	1.64 ± 0.92	0.0736	1.81 ± 1.50	0.2266
Age	0.07 ± 0.04	0.1016	0.10 ± 0.04	**0.0108**	−0.003 ± 0.06	0.9531
IM (3) vs. IM (0–2)	2.48 ± 3.16	0.4324	1.12 ± 1.89	0.5527	1.95 ± 2.23	0.3808
Atrophy (3) vs. atrophy (0–2)	−1.26 ± 2.03	0.5326	−1.71 ± 1.53	0.2661	−1.25 ± 1.28	0.3297
**B. Validation set 2**						
**Risk factors**	**miR-26a**		**miR-375**		**miR-1260**	
	**β coefficient**	***P*-value**	**β coefficient**	***P*-value**	**β coefficient**	***P*-value**
GC patients vs. healthy volunteers	−5.34 ± 0.99	**<0.0001**	−6.82 ± 1.12	**<0.0001**	−9.00 ± 1.36	**<0.0001**
M vs. F	0.60 ± 0.64	0.35	1.17 ± 0.89	0.1859	1.62 ± 1.19	0.1734
Age	0.05 ± 0.02	**0.0421**	0.11 ± 0.05	**0.0172**	0.03 ± 0.05	0.5294
IM (3) vs. IM (0–2)	0.10 ± 0.37	0.7834	1.07 ± 1.54	0.4889	1.78 ± 2.05	0.3866
Atrophy (3) vs. atrophy (0–2)	−2.10 ± 1.07	**0.0497**	−4.27 ± 2.34	0.0675	−3.44 ± 2.15	0.1095

GC: gastric carcinoma; IM: intestinal metaplasia.

**Figure 1:**
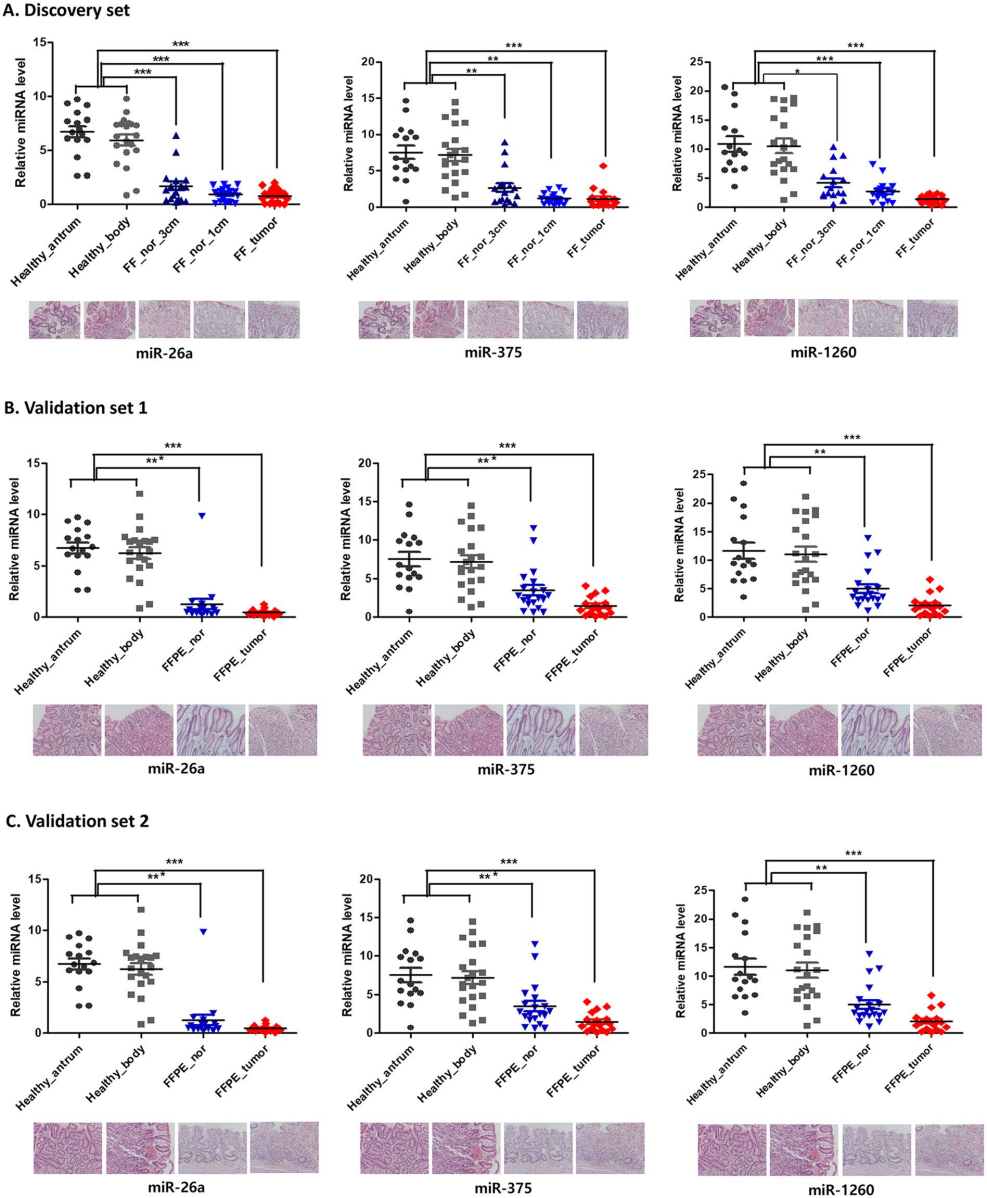
Comparison between relative miRNA levels of gastric mucosae from healthy volunteers and non-tumor tissues from gastric cancer patients by multivariate analysis in training (**A**) and validation sets (**B**,**C**). ^***^P < 0.0001, ^**^P < 0.005, ^*^P < 0.05.

The miRNA expression levels stratified by location within the stomach and by specimen type (frozen versus FFPE) are precisely described in Fig. 1. However, miR-107, miR-300, and miR-370 did not show any significant difference in miRNA expression in all samples (Supplementary Table 1).

### Dysregulated miRNA expression within non-tumor and tumor in gastric cancer patientsthe training set, there was a significant difference in miR-375

In (*P* = 0.0342) and miR-1260 (*P* < 0.0001), but not miR-26a (*P* = 0.2288) when comparing miRNA expression in the gastric mucosa 1 cm apart from the tumor and at the tumor itself. miRNA expression was significantly different between normal mucosa 3 cm away from the tumor and at the tumor (miR-26a, *P* = 0.0342; miR-375, *P* = 0.001; and miR-1260, *P* < 0.0001) (Supplementary Fig. 1). In validation set 1, there was a significant difference in miR-375 (*P* = 0.006) and miR-1260 (*P* = 0.0003), but not miR-26a (*P* = 0.0654). In validation set 2, there was a significant difference in miR-1260 (*P* = 0.0054), but not miR-26a (*P* = 0.0783) and miR-375 (*P* = 0.1054).

### Comparison of miRNA expression according to the number of tumors and Lauren’s classification

The miRNA expression was compared between non-tumor tissues and between tumor tissues according to the number of tumors and Lauren’s classification, respectively. In comparison between non-tumor tissues, miR-1260 expression in multiple GCs was significantly lower than that in solitary GC (*P* = 0.0289), but there was no significant difference in miR-26a (*P* = 0.8928) and miR-375 (*P* = 0.4007). When miRNA expression was compared within non-tumor tissues according to Lauren’s classification, there were no significant variables with a small number of cases in univariate analysis of all miRNAs.

In the comparison between tumor tissues, there was a statistically significant difference in all miRNAs except for miR-26a between solitary GC and multiple GCs. The miR-375 and miR-1260 expression of multiple GCs was significantly lower than that of solitary GC (miR-26a, *P* = 0.369; miR-375, *P* = 0.0017; and miR-1260, *P* = 0.0068). The difference in miRNA expression between intestinal type and diffuse type within tumor tissues was not statistically significant in most cases. In a training set, only miR-26a expression showed a significant difference between intestinal and diffuse types (*P* = 0.0078), and expression of the other miRNAs showed no significant difference according to Lauren’s classification (miR-375 and miR-1260, *P* = 0.7209). In validation set 1, there were no significant differences in miRNA expression according to Lauren’s classification (miR-26a, *P* = 0.0557; miR-375, *P* = 0.4117; and miR-1260, *P* > 0.999). In validation set 2, miRNAs showed similar results to validation set 1 (miR-26a, *P* = 0.0835; miR-375, *P* = 0.989; and miR-1260, *P* = 0.2411).

### Predictability of miRNA expression and differential expression of target genes of these miRNAs

**Figure 2:**
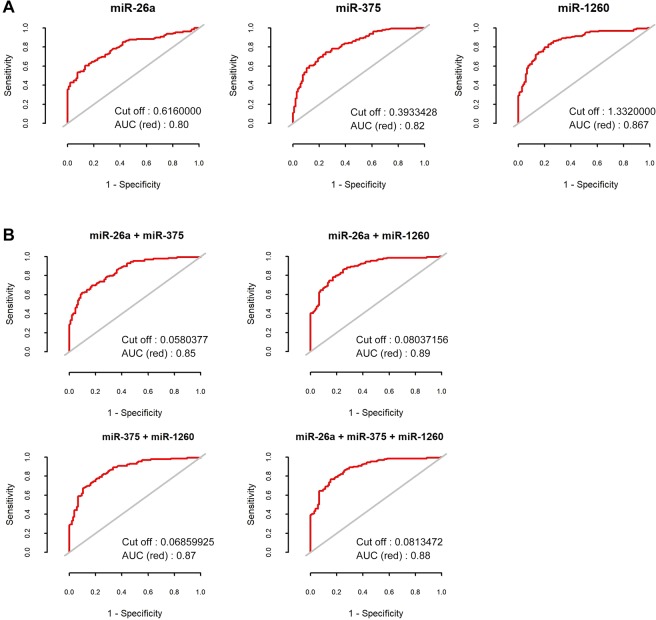
Receiver operator characteristic (ROC) curves of miR-26a, miR-375, and miR-1260 (**A**). ROC curves of combination of these miRNA expressions with linear regression model (**B**).

To evaluate the predictability of miRNA for cancer-prone environments, ROC curves were generated. The AUCs of three miRNAs were over 0.8. When these miRNA expressions were combined in different combinations, the combination of miR-26a and miR-1260 had the highest AUC value of 0.89. ROC curves with AUC values and cut-off values were described in Fig. 2.

**Figure 3:**
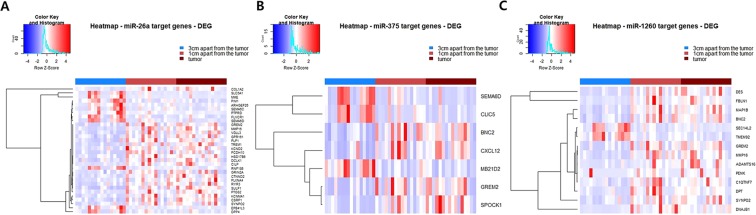
Heatmaps of differentially expressed target genes of miR-26a (**A**), miR-375 (**B**), and miR-1260 (**C**).

Genes that were differentially expressed in tumor and non-tumor mucosa located 3 cm and 1 cm apart from the tumor were analyzed using transcriptome sequencing data. Among differentially expressed genes, potential target genes of miR-26a, miR-375, and miR-1260 were selected, and heatmaps of differentially expressed target genes are depicted in Fig. 3.

According to fold changes of expression level between non-tumor and tumor, representative target genes were *CLIP2, TREM1* (miR-26a), *SPOCK1* (miR-375), *PENK*, and *ADAMTS16* (miR-1260). *GREM2* was the common target gene of the three miRNAs, and *MMP16* and *SYNPO2* were the common target genes of miR-26a and miR-1260.

## Discussion

In the era of precision medicine for cancer prevention and early diagnosis, it is important to identify early molecular events and clinically applicable biomarkers^1^. miRNA is involved in gastric carcinogenesis^2^; however, its relationship with cancer-prone environment, an microenvironment where histopathology is within normal but accompanied by genetic or epigenetic alterations that cannot be visible and associated with susceptibility to malignant change, is not clearly demonstrated. In the present study, we tested expression of 6 miRNAs in 201 gastric mucosal tissues from 74 patients with GC and 37 gastric tissues from 20 healthy volunteers by qRT-PCR. We found that dysregulated miRNA is involved in early non-neoplastic gastric mucosa and can be used as a biomarker to distinguish cancer-prone environments.

In this study, expression of three miRNAs from normal mucosa of healthy volunteers was significantly higher than that of non-tumor mucosa from patients with GC in a training set and two validation sets. This result suggests that miRNA alterations are present in cancer-prone environments and are involved in gastric carcinogenesis. Link *et al*.^3^ reported that miR-21, miR-155, and miR-223 expression patterns gradually increase with progression of Correa’s cascade by analyzing miRNA expression in gastric mucosa from patients with chronic non-atrophic gastritis, atrophic gastritis, and GC compared to normal controls. Another study showed miRNA expression in non-cancerous tissue from gastritis, adjacent normal tissue, and GC tumor to show that miR-10a, miR-21, and miR-135b expression gradually increased from non-cancerous tissue to tumor tissue^4^. Although methylation levels of miRNA genes were explored, direct comparison of miRNA in non-tumorous gastric mucosa and healthy volunteers has not been performed^20,21^. Based on our comparison study and previous observations, we speculate that changes of miRNA expression start from cancer-prone environments. To prove this, heatmaps of differentially expressed genes were generated using RNA sequencing data to demonstrate that target genes of those miRNAs are significantly dysregulated. *TREM1* and *CLIP2* are representative target genes of miR-26a. *TREM-1* is a known trigger of innate immunity that stimulates secretion of tumor necrosis factor-alpha (TNF-α) and interlukin-8 (IL-8)^22,23^. Gastric epithelial *TREM-1* expression was up-regulated directly by *H. pylori* and amplifies inflammation by up-regulation of IL-8^24^. Based on previous observations, we can infer that *TREM-1* will be involved in cancer-prone environments of *H. pylori* infection and active inflammation. *CLIP2* involves the TNF-α singling pathway^25^ to play an important role in gastric tumorigenesis^26^. *SPOCK1*, a representative target gene of miR-375, has been demonstrated to facilitate metastasis in certain types of cancer and to promote GC invasion and metastasis through a Slug-dependent mechanism^27^. *PENK* and *ADAMTS16* are representative DEGs of miR-1260. *PENK* is down-regulated and methylated in prostate, bladder, pancreatic, and colorectal cancers^28^; however, its relationship in GC has not been explored. The ADAMTS family is thought to play an important role in carcinogenesis; however, the function and regulation of *ADAMTS16* in carcinogenesis are not clearly defined^29^.

In the training set, miRNAs expression in non-tumor mucosa was significantly different based on distance from tumor. The expression level of non-tumor mucosa obtained at 1 cm distance is similar to that of tumor and non-tumor mucosa obtained at 3 cm distance, which is also similar to that of healthy volunteers. These results suggest that a cancer-prone environment was already formed in the normal mucosa close to the tumor. The possibility of recurrence can be reduced by ensuring resection margins of at least 1 cm when an ESD procedure is performed. However, more data is needed to provide guidelines for accurate resection margins. Only miR-1260 showed differences in expression between non-tumor and tumor tissues in validation sets 1 and 2, whereas miR-26a did not show expression differences between non-tumor and tumor tissues in all validation sets.

Additionally, to identify if multiple GCs are more frequent in the background of cancer-prone environments, miRNA expression in non-tumorous gastric mucosae from patients with single GC and multiple GC was compared. miR-1260 expression was significantly lower in non-tumorous gastric tissues of patients with multiple GC than solitary GC (*P* = 0.0289), although miR-26a (*P* = 0.8928) and miR-375 (*P* = 0.4007) did not show any significant difference. Previous studies showed that the methylation level of miR-34b/c in non-cancerous gastric mucosa from multiple GCs, not solitary GC, was significantly higher than that of healthy controls. As a result, miR-34b/c was suggested as a predictive marker for recurrence and metachronous GC risk^21,30^. Our results also strengthen the importance of miRNA expression as a biomarker.

In the present study, no difference in miRNA expression was found within intestinal and diffuse type tumors except for miR-26a. Although intestinal type and diffuse type are thought to be caused by distinct mechanisms^31,32^, *H. pylori* infection is related to gastric carcinogenesis in both Lauren types^33^, and *H. pylori* infection is known to be associated with miRNA expression patterns^3,20,21,34^. Given these observations, we hypothesize that *H. pylori* and miRNA expression may be associated with field cancerization regardless of Lauren’s classification. In the present study, lower miR-26a expression was significantly associated with diffuse type GC, but the relationship between miR-26a and Lauren’s classification is controversial. While higher miR-26a expression was reported to be associated with diffuse type GCs^35^, others reported downregulated miR-26a in poorly differentiated GCs^36^. More research is needed to clarify how miRNA functions in tumorigenesis according to Lauren’s classification.

In the present study, the AUC of all 3 miRNAs was over 0.8. The combination of miR-26a and miR-1260 showed the highest AUC value of 0.89, suggesting that combination of the two miRNAs can be used as a biomarker to predict cancer-prone environments. Furthermore, if a patient has a very small tumor and the biopsy is likely to fail, measurement of miRNA expression may aid in clinical decision making regarding treatment or follow-up. Further prospective clinical trials are needed to confirm our results.

Our study has several limitations. First, the sample size of the training set was small (18 patients, 54 samples). To overcome this limitation, results were validated with FFPE samples from independent cohorts (56 patients, 147 samples). Second, the age and sex distribution of healthy volunteers was quite different from that of patients with GC. To overcome this limitation, multivariate analysis was used to correct for effects of other factors such as age, sex, and degrees of intestinal metaplasia and atrophy. In some cases, miRNA expression decreased with age, suggesting that dysregulated miRNA expression is related to age; however, even with age, the difference in miRNA expression between normal gastric tissues in healthy volunteers and non-tumorous tissue in GC was encouraging. Third, we do not demonstrate predictability of miRNA with clinical follow-up data because of limited time and instead, calculated AUC of the miRNAs to measure predictability. Fourth, to address the link between miRNAs and target genes, more strong experimental evidences are strongly recommended. Further studies are recommended to prove our hypothesis.

In conclusion, dysregulated miRNA expression occurs in non-neoplastic gastric mucosa adjacent to tumors. Combined dysregulated miRNAs can be used as a biomarker to predict cancer-prone environments and stratify follow-up for patients with GC.

## Materials and Methods

### Ethics statement

Tumor and non-tumor samples were obtained from patients with GC, and normal samples were obtained from healthy volunteers at Samsung Comprehensive Cancer Center. Informed consent was obtained from all individuals who participated in this study. The study protocol was approved by the institutional review board of Samsung Medical Center (IRB 2017-12-087-009), and all experiments were performed in accordance with approved guidelines and regulations.

### Samples

Fresh samples were collected for the training set. Fresh tumor samples were obtained from gastrectomy or endoscopic submucosal dissection specimens of 18 patients. Fresh non-tumor samples were also obtained at 3 cm and 1 cm apart from the tumor of the same specimens in parallel. All fresh samples were snap frozen in liquid nitrogen immediately after collection and stored at −80 °C until use.

Formalin-fixed paraffin-embedded (FFPE) samples were collected for validation sets 1 and 2. FFPE tumor and non-tumor samples of 19 patients diagnosed with solitary GC were collected for validation set 1, and FFPE tumor and non-tumor samples of 37 patients diagnosed with multiple GCs were collected for validation set 2. Only one tumor sample was collected from two patients who were diagnosed with multiple GCs. FFPE tumor and non-tumor tissues were obtained from gastrectomy specimens (Supplementary Fig. 2). All patients had no prior chemo- or radiation therapy.

**Figure 4:**
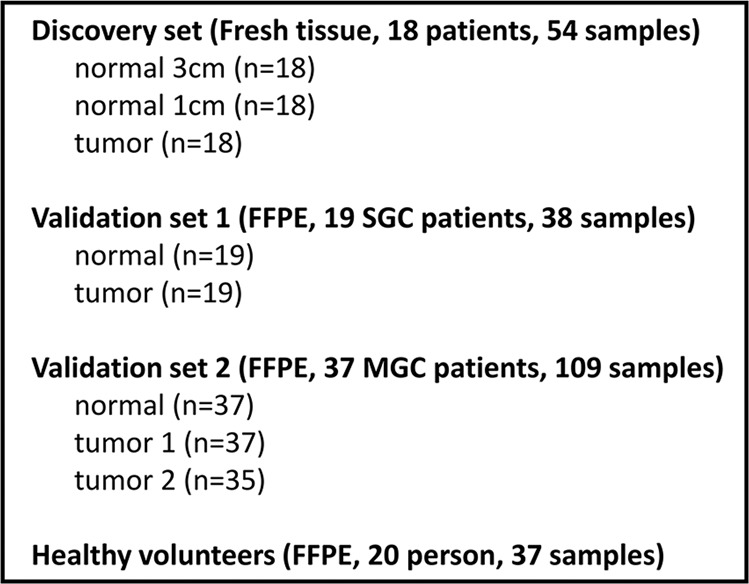
Number of patients and samples used in the study. FFPE: Formalin-fixed paraffin-embedded; SGC: solitary gastric carcinoma; MGC: multiple gastric carcinoma.

In addition, samples of 37 normal gastric mucosae were collected from endoscopic biopsy specimens of 20 healthy volunteers who had not been diagnosed with any type of cancer. The histology of all tissue samples was confirmed before miRNAs extraction. The Lauren’s type of the tumor was also reviewed, and the degree of intestinal metaplasia and atrophy in non-tumor samples from GC patients and normal samples from healthy volunteers were scored based on Sydney classification. *Helicobacter pylori* status was determined by serology or histology. Cases with positive serology and/or positive histology were defined as *H. pylori-*positive. Cases with no direct detection of *H. pylori* in serology or histology were defined as *H. pylori-*negative. The number of patients and samples used in this study is described in Fig. 4.

### Clinicopathologic characteristics of samples

In total, 201 gastric mucosal tissues from 74 patients with GC and 37 gastric mucosal tissues from 20 healthy volunteers were used. In the training set, median age was 66, and the male to female ratio was 13:5. The number of intestinal type cases was 14 (77.8%). The number of cases with *H. pylori* infection was 8 (42.1%). All but two cases had early gastric cancer (Supplementary Table 2). In validation set 1, the median age was 65, and the male to female ratio was 13:6. The number of intestinal type cases was 15 (78.9%). The number of cases with *H. pylori* infection was 10 (52.6%). All cases were early gastric cancer (Supplementary Table 3). In validation set 2, the median age was 61, and the male to female ratio was 24:13. The number of intestinal type cases was 54 (75%). The number of cases with *H. pylori* infection was 26 (70.3%). All but seven cases represented early gastric cancer (Supplementary Table 4). In healthy volunteers, the median age was 43, and the male to female ratio was 8:12. The number of cases with *H. pylori* infection was 9 (45%; Supplementary Table 5). The clinicopathologic characteristics of study cases are described in Table 3.

**Table 3:** The clinicopathologic characteristics of the study cases.

	Training	Validation 1	Validation 2	Healthy
**Age (median, range)**	66 (43–82)	65 (49–83)	61 (40–84)	43 (25–52)
**Sex**				
Male	13 (72.2%)	13 (68.4%)	24 (64.9%)	8 (40%)
Female	5 (27.8%)	6 (31.6%)	13 (35.1%)	12 (60%)
**Lauren Classification**				
Intestinal	14 (77.8%)	15 (78.9%)	54 (75%)	
Diffuse	4 (22.2%)	4 (21.1%)	18 (25%)	
**H. Pylori**				
Positive	10 (55.6%)	8 (42.1%)	26 (70.3%)	9 (45%)
Negative	8 (44.4%)	11 (57.9%)	11 (29.7%)	11 (55%)
**Intestinal metaplasia**				
Present	22 (61.1%)	18 (94.7%)	22 (59.5%)	31 (77.5%)
Negative	14 (38.9%)	1 (5.3%)	15 (40.5%)	6 (22.5%)
**Atrophy**				
Present	25 (69.4%)	16 (84.2%)	23 (62.2%)	21 (56.8%)
Negative	11 (30.6%)	3 (15.8%)	14 (37.8%)	16 (43.2%)
**Region**				
Antrum	11 (61.1%)	10 (52.6%)	26 (36.1%)	15 (40.5%)
Body	6 (33.3%)	7 (36.9%)	41 (57%)	19 (51.4%)
Others	1 (5.6%)	2 (10.5%)	5 (6.9%)	3 (8.1%)
**Stage**				
Early gastric carcinoma	16 (88.9%)	19 (100%)	30 (81.1%)	
Advanced gastric carcinoma	2 (11.1%)		7 (18.9%)	

### Quantitative real-time PCR analysis (qRT-PCR)

Six miRNAs were selected based on our previous paper^2^. Three of them (miR-26a, miR-375, and miR-1260) were expressed highly in normal tissues and 3 of them (miR-107, miR-300, and miR-370) were expressed highly in GCs. Ten serial paraffin cuts were obtained in an Eppendorf tube and deparaffinized in xylene. Total RNA was isolated from FFPE and fresh frozen samples using RNeasy Micro Kit (Qiagen, Hilden, Germany) according to the manufacturer’s instructions. RNA concentrations were measured using NanoDrop (Thermo Fisher Scientific, Wilmington, DE, USA). Total RNA from each sample was reverse transcribed with the TaqMan MicroRNA Reverse Transcription kit (Thermo Fisher Scientific, Wilmington, DE, USA). Reverse transcription was performed with the following thermal cycling parameters: 30 minutes at 16 °C, 30 minutes at 42 °C, and 5 minutes at 85 °C (BioRad, Hercules, CA, USA).

miRNA expression was determined with TaqMan MicroRNA primer/probe sets. All qPCR reactions were performed with the 7900 Fast Real-Time PCR System (Applied Biosystems, Foster City, CA, USA). Gene expressions for hsa-miR-375 (Assay ID, 000564), hsa-miR-370 (Assay ID, 002275), hsa-miR-26a (Assay ID, 000405), hsa-miR-300 (Assay ID, 241035), hsa-miR-1260 (Assay ID, 002896), and hsa-miR-107 (Assay ID, 000443) were quantified by TaqMan microRNA Assays (Applied Biosystems, Foster City, CA, USA) according to the manufacturer’s protocol and normalized by U6 snRNA (Assay ID 001973). PCR amplification of target genes and quantification of the amount of PCR product were performed by ABI PRISM 7900 HT Sequence Detection System (Applied Biosystems, Foster City, CA, USA). Differences in expression were determined by relative quantification method; Ct values of the test genes were normalized to Ct values of endogenous control U6 snRNA. The fold change was calculated using the equation 2-ΔΔCt. To exclude the technical bias, we conducted the experiments in triplicate and at the same way and at the same time in each cohort.

### Receiver operating characteristic (ROC) curve and area under the curve (AUC)

To evaluate miRNA as a predictive biomarker, receiver operating characteristic (ROC) curve and area under the curve (AUC) analysis were performed with ROCR package in R software https://www.r-project.org/ (version 3.4.4). Expression values of each miRNA were used to evaluate the biomarker potential of each miRNA. To evaluate combined miRNA, the score of linear regression model of 2 or 3 miRNA was used. All analyses were performed in R software (version 3.4.4).

### RNA sequencing for analysis of target genes of miRNA

For RNA sequencing, after RNA extraction, only qualified samples proceeded to library construction using a TruSeq RNA Access Library Prep Kit (Illumina, Inc., San Diego, CA, USA). The average length of each read was ~101 bp, and libraries were sequenced on an Illumina platform following the manufacturer’s instructions. After generating raw data in FASTQ format, preprocessing was performed using DESeq2 package in R software (version 3.4.4). Count-based matrix data were generated.

To search for target genes of each miRNA, the public database miRDB (http://mirdb.org/index.html) was used^37^. To investigate the expression pattern in target genes of each miRNA, heatmaps were generated using RNA sequencing data from 48 samples of the training sample set. Differentially expressed gene (DEG) analysis was also performed using DESeq. 2 packages to identify DEGs among the target genes. Heatmaps were generated using expression values of DEGs among target genes.

### Statistical analysis

To compare miRNA expression in non-tumor samples from GC patients with those of normal samples from healthy volunteers, a generalized estimating equation (GEE) was used. To compare miRNA expression between non-tumor and tumor, Wilcoxon signed rank test was used for the training set and GEE was used for validation sets. To identify differences in miRNA expression according to the number of tumors, linear regression analysis was used in non-tumor samples and GEE was used in tumor samples. To identify differences in miRNA expression according to Lauren’s classification, Linear regression analysis and GEE were used in non-tumor samples, and Exact Wilcoxon Two-Sample Test and GEE were used in tumor samples. A *P*-value < 0.05 was interpreted as significant. All statistical analyses were executed using SAS version 9.4 (SAS Institute Inc., Cary, NC, USA).

## Supplementary information

Supplementary Information.

Supplementary Information 2.

Supplementary Information 3.

Supplementary Information 4.
