# Supplementary figures and images for: Dysregulated miRNA in a cancer-prone environment: A study of gastric non-neoplastic mucosa
Source: Sci Rep. 2020 Apr 20;10:6600. doi: 10.1038/s41598-020-63230-1 (PMC7171080; doi:10.1038/s41598-020-63230-1)

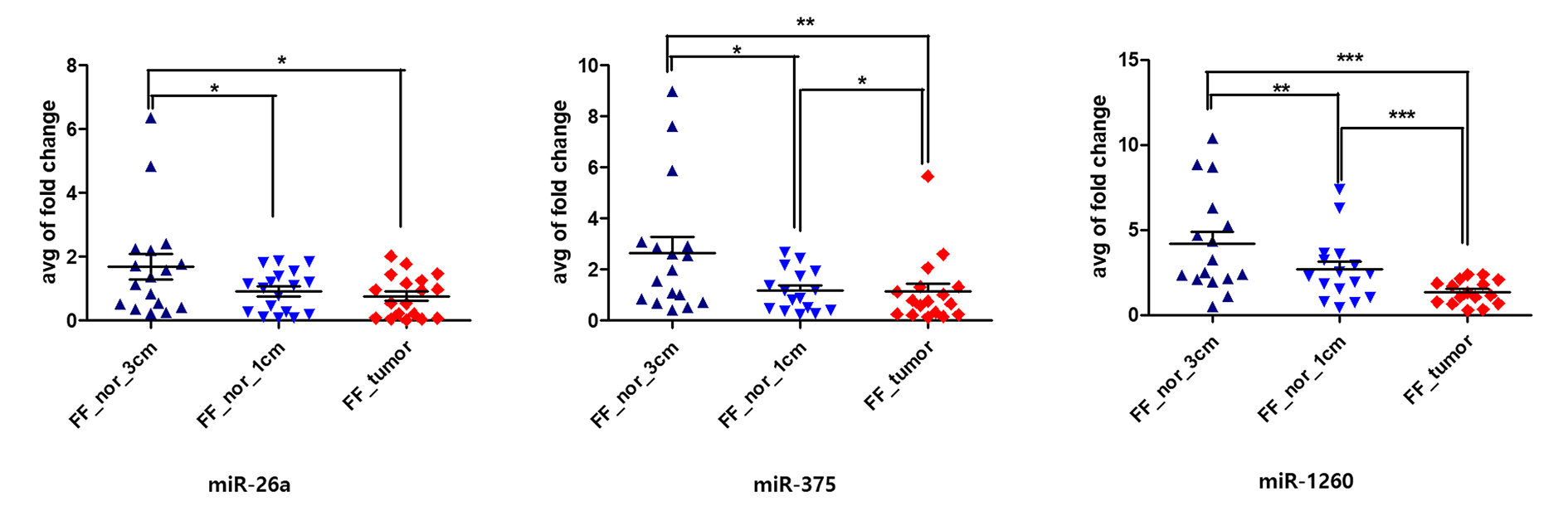

Supplement: Supplementary file 1 — Supplementary Information. [file 41598_2020_63230_MOESM1_ESM.tif]

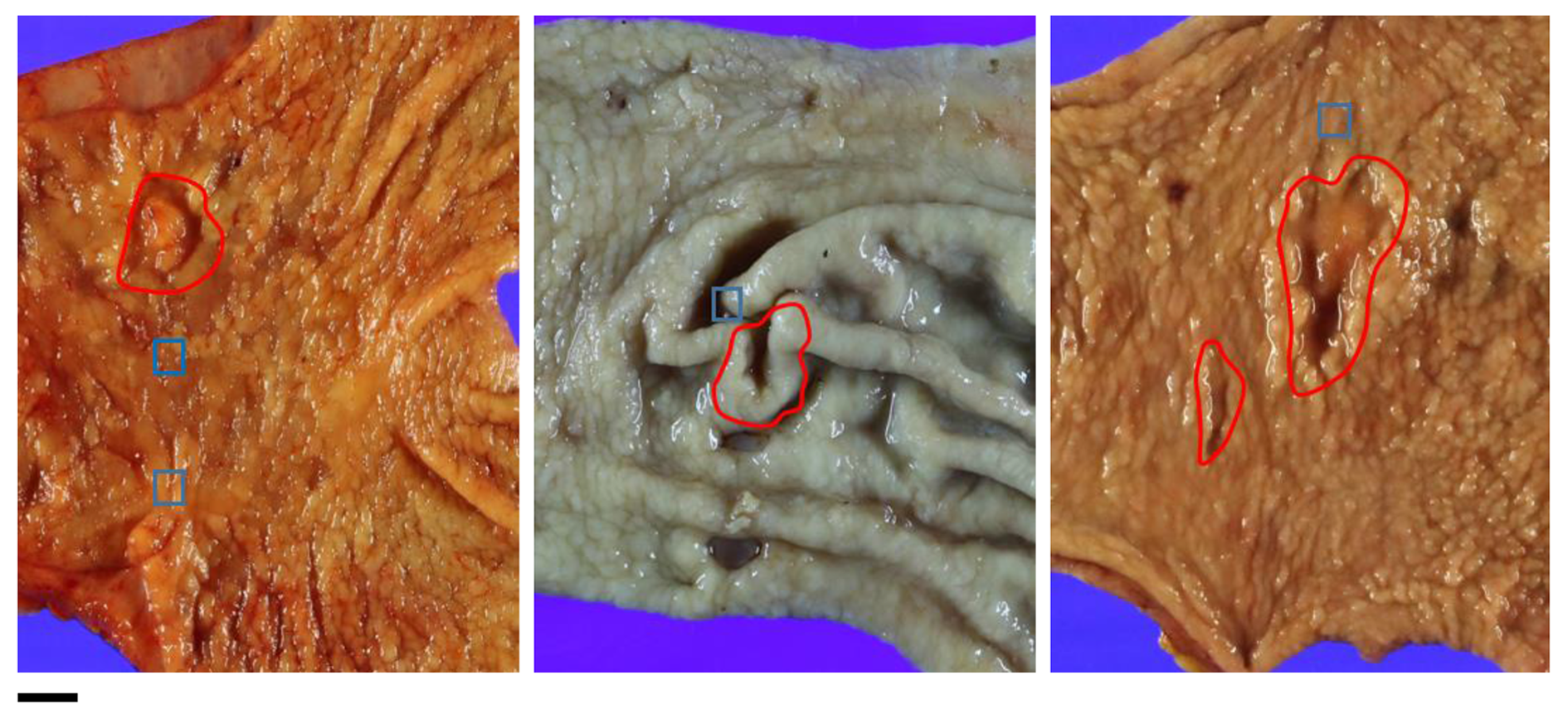

Supplement: Supplementary file 2 — Supplementary Information 2. [file 41598_2020_63230_MOESM2_ESM.tif]
