# Supplementary material for: Dysregulated miRNA in a cancer-prone environment: A study of gastric non-neoplastic mucosa
Source: Sci Rep. 2020 Apr 20;10:6600. doi: 10.1038/s41598-020-63230-1 (PMC7171080; doi:10.1038/s41598-020-63230-1)
Supplement: Supplementary file 3 — Supplementary Information 3. [file 41598_2020_63230_MOESM3_ESM.docx]

**Supplementary Table 1. The levels of miR-107, miR-300, and miR-370 in non-neoplastic mucosae and tumors**

| **No.** | **Sex** | **Age** | **Status** | **Lauren's class** | **miR-107** | **miR-300** | **miR-370** | **IM** | **Atrophy** | **H.pylori** | **AJCC stage** |
| --- | --- | --- | --- | --- | --- | --- | --- | --- | --- | --- | --- |
| 1 | M | 63 | Tumor_1 | intestinal | 0.000 | 0.000 | 0.001 |  |  | Positive | T1b N0 |
|  |  |  | Tumor_2 | intestinal | 0.000 | 0.000 | 0.001 |  |  |  |  |
|  |  |  | Normal |  | 0.002 | 0.038 | 0.467 | 1 | 1 |  |  |
| 2 | M | 48 | Tumor_1 | diffuse | 0.000 | 0.000 | 0.001 |  |  | Negative | T1b N0 |
|  |  |  | Tumor_2 | diffuse | 0.000 | 0.000 | 0.000 |  |  |  |  |
|  |  |  | Normal |  | 0.000 | 0.000 | 0.001 | 0 | 0 |  |  |
| 3 | F | 48 | Tumor_1 | diffuse | 0.000 | 0.000 | 0.001 |  |  | Positive | T1b N0 |
|  |  |  | Tumor_2 | diffuse | 0.000 | 0.000 | 0.001 |  |  |  |  |
|  |  |  | Normal |  | 0.000 | 0.000 | 0.001 | 1 | 0 |  |  |
| 4 | F | 68 | Tumor_1 | diffuse | 0.000 | 0.000 | 0.000 |  |  | Negative | T2 N1 |
|  |  |  | Tumor_2 | diffuse | 0.000 | 0.000 | 0.000 |  |  |  |  |
|  |  |  | Normal |  | 0.000 | 0.000 | 0.001 | 0 | 2 |  |  |
| 5 | M | 45 | Tumor_1 | intestinal | 0.000 | 0.000 | 0.000 |  |  | Positive | T1b N0 |
|  |  |  | Tumor_2 | intestinal | 0.001 | 0.004 | 0.017 |  |  |  |  |
|  |  |  | Normal |  | 0.000 | 0.000 | 0.001 | 0 | 0 |  |  |
| 6 | M | 64 | Tumor_1 | intestinal | 0.001 | 0.000 | 0.011 |  |  | Negative | T1b N1 |
|  |  |  | Tumor_2 | intestinal | 0.001 | 0.000 | 0.000 |  |  |  |  |
|  |  |  | Normal |  | 0.000 | 0.000 | 0.001 | 3 | 3 |  |  |
| 7 | F | 60 | Tumor_1 | intestinal | 0.000 | 0.000 | 0.000 |  |  | Positive | T1a N0 |
|  |  |  | Tumor_2 | diffuse | 0.001 | 0.000 | 0.000 |  |  |  |  |
|  |  |  | Normal |  | 0.000 | 0.000 | 0.000 | 3 | 3 |  |  |
| 8 | F | 42 | Tumor_1 | diffuse | 0.000 | 0.000 | 0.000 |  |  | Positive | T1b N0 |
|  |  |  | Tumor_2 | diffuse | 0.000 | 0.000 | 0.004 |  |  |  |  |
|  |  |  | Normal |  | 0.000 | 0.000 | 0.001 | 1 | 2 |  |  |
| 9 | M | 79 | Tumor_1 | intestinal | 0.000 | 0.000 | 0.000 |  |  | Positive | T1b N1 |
|  |  |  | Tumor_2 | intestinal | 0.000 | 0.000 | 0.000 |  |  |  |  |
|  |  |  | Normal |  | 0.000 | 0.000 | 0.000 | 3 | 3 |  |  |
| 10 | M | 66 | Tumor_1 | intestinal | 0.002 | 0.000 | 0.002 |  |  | Negative | T1a N0 |
|  |  |  | Tumor_2 | intestinal | 0.002 | 0.000 | 0.001 |  |  |  |  |
|  |  |  | Normal |  | 0.001 | 0.000 | 0.001 | 3 | 3 |  |  |
| 11 | M | 55 | Tumor_1 | intestinal | 0.000 | 0.000 | 0.000 |  |  | Negative | T1b N0 |
|  |  |  | Tumor_2 | intestinal | 0.000 | 0.000 | 0.000 |  |  |  |  |
|  |  |  | Normal |  | 0.000 | 0.000 | 0.001 | 3 | 3 |  |  |
| 12 | F | 52 | Tumor_1 | intestinal | 0.000 | 0.000 | 0.000 |  |  | Positive | T1b N0 |
|  |  |  | Tumor_2 | diffuse | 0.000 | 0.000 | 0.001 |  |  |  |  |
|  |  |  | Normal |  | 0.001 | 0.000 | 0.002 | 1 | 1 |  |  |
| 13 | M | 70 | Tumor_1 | intestinal | 0.000 | 0.000 | 0.001 |  |  | Negative | T1b N0 |
|  |  |  | Tumor_2 | intestinal | 0.000 | 0.000 | 0.001 |  |  |  |  |
|  |  |  | Normal |  | 0.001 | 0.000 | 0.002 | 3 | 3 |  |  |
| 14 | M | 63 | Tumor_1 | intestinal | 0.000 | 0.000 | 0.000 |  |  | Positive | T1a N0 |
|  |  |  | Tumor_2 | intestinal | 0.000 | 0.000 | 0.000 |  |  |  |  |
|  |  |  | Normal |  | 0.003 | 0.001 | 0.005 | 0 | 0 |  |  |
| 15 | F | 51 | Tumor_1 | diffuse | 0.000 | 0.000 | 0.001 |  |  | Positive | T1a N0 |
|  |  |  | Tumor_2 | diffuse | 0.000 | 0.000 | 0.001 |  |  |  |  |
|  |  |  | Normal |  | 0.000 | 0.000 | 0.000 | 0 | 0 |  |  |
| 16 | M | 61 | Tumor_1 | intestinal | 0.002 | 0.000 | 0.001 |  |  | Positive | T1b N0 |
|  |  |  | Tumor_2 | intestinal | 0.001 | 0.000 | 0.000 |  |  |  |  |
|  |  |  | Normal |  | 0.001 | 0.000 | 0.001 | 0 | 0 |  |  |
| 17 | F | 46 | Tumor_1 | diffuse | 0.000 | 0.000 | 0.001 |  |  | Positive | T1b N1 |
|  |  |  | Tumor_2 | diffuse | 0.000 | 0.000 | 0.001 |  |  |  |  |
|  |  |  | Normal |  | 0.022 | 0.007 | 0.044 | 0 | 0 |  |  |
| 18 | M | 60 | Tumor_1 | intestinal | 0.001 | 0.000 | 0.001 |  |  | Positive | T1a N0 |
|  |  |  | Tumor_2 | intestinal | 0.001 | 0.000 | 0.001 |  |  |  |  |
|  |  |  | Normal |  | 0.001 | 0.000 | 0.001 | 3 | 3 |  |  |
| 19 | M | 69 | Tumor_1 | intestinal | 0.000 | 0.000 | 0.000 |  |  | Positive | T1a N0 |
|  |  |  | Tumor_2 | intestinal | 0.001 | 0.000 | 0.001 |  |  |  |  |
|  |  |  | Normal |  | 0.001 | 0.000 | 0.002 | 0 | 3 |  |  |
| 20 | F | 71 | Tumor_1 | intestinal | 0.001 | 0.000 | 0.001 |  |  | Positive | T2 N1 |
|  |  |  | Tumor_2 | intestinal | 0.001 | 0.000 | 0.001 |  |  |  |  |
|  |  |  | Normal |  | 0.001 | 0.000 | 0.001 | 2 | 3 |  |  |
| 21 | M | 67 | Tumor_1 | intestinal | 0.001 | 0.000 | 0.001 |  |  | Positive | T2 N3a |
|  |  |  | Tumor_2 | intestinal | 0.001 | 0.000 | 0.001 |  |  |  |  |
|  |  |  | Normal |  | 0.001 | 0.000 | 0.004 | 3 | 3 |  |  |
| 22 | M | 71 | Tumor_1 | intestinal | 0.001 | 0.000 | 0.001 |  |  | Positive | T1b N0 |
|  |  |  | Tumor_2 | intestinal | 0.001 | 0.000 | 0.001 |  |  |  |  |
|  |  |  | Normal |  | 0.001 | 0.000 | 0.001 | 3 | 3 |  |  |
| 23 | M | 65 | Tumor_1 | intestinal | 0.001 | 0.000 | 0.001 |  |  | Positive | T1b N0 |
|  |  |  | Tumor_2 | intestinal | 0.001 | 0.000 | 0.001 |  |  |  |  |
|  |  |  | Normal |  | 0.003 | 0.000 | 0.002 | 2 | 2 |  |  |
| 24 | M | 44 | Tumor_1 | intestinal | 0.000 | 0.000 | 0.000 |  |  | Positive | T1a N0 |
|  |  |  | Tumor_2 | intestinal | 0.000 | 0.000 | 0.000 |  |  |  |  |
|  |  |  | Normal |  | 0.000 | 0.000 | 0.001 | 0 | 0 |  |  |
| 25 | F | 58 | Tumor_1 | intestinal | 0.000 | 0.000 | 0.000 |  |  | Positive | T1a N0 |
|  |  |  | Tumor_2 | intestinal | 0.000 | 0.000 | 0.000 |  |  |  |  |
|  |  |  | Normal |  | 0.000 | 0.000 | 0.000 | 3 | 3 |  |  |
| 26 | M | 61 | Tumor_1 | diffuse | 0.000 | 0.000 | 0.000 |  |  | Positive | T1b N0 |
|  |  |  | Tumor_2 | intestinal | 0.000 | 0.000 | 0.000 |  |  |  |  |
|  |  |  | Normal |  | 0.001 | 0.000 | 0.002 | 0 | 2 |  |  |
| 27 | M | 69 | Tumor_1 | intestinal | 0.000 | 0.000 | 0.000 |  |  | Negative | T1b N0 |
|  |  |  | Tumor_2 | intestinal | 0.000 | 0.000 | 0.000 |  |  |  |  |
|  |  |  | Normal |  | 0.000 | 0.000 | 0.001 | 3 | 3 |  |  |
| 28 | M | 57 | Tumor_1 | intestinal | 0.000 | 0.000 | 0.000 |  |  | Negative | T1a N0 |
|  |  |  | Tumor_2 | intestinal | 0.000 | 0.000 | 0.000 |  |  |  |  |
|  |  |  | Normal |  | 0.071 | 0.025 | 0.380 | 0 | 0 |  |  |
| 29 | M | 52 | Tumor_1 | intestinal | 0.000 | 0.000 | 0.000 |  |  | Positive | T1b N0 |
|  |  |  | Tumor_2 | intestinal | 0.000 | 0.000 | 0.000 |  |  |  |  |
|  |  |  | Normal |  | 0.001 | 0.000 | 0.001 | 1 | 0 |  |  |
| 30* | F | 40 | Tumor | diffuse | 0.001 | 0.000 | 0.001 |  |  | Positive | T2 N0 |
|  |  |  | Normal |  | 0.000 | 0.000 | 0.000 | 0 | 0 |  |  |
| 31 | F | 57 | Tumor_1 | diffuse | 0.000 | 0.000 | 0.001 |  |  | Negative | T1a N0 |
|  |  |  | Tumor_2 | diffuse | 0.000 | 0.000 | 0.000 |  |  |  |  |
|  |  |  | Normal |  | 0.000 | 0.000 | 0.001 | 0 | 0 |  |  |
| 32 | M | 64 | Tumor_1 | intestinal | 0.000 | 0.000 | 0.000 |  |  | Positive | T2 N1 |
|  |  |  | Tumor_2 | intestinal | 0.000 | 0.000 | 0.001 |  |  |  |  |
|  |  |  | Normal |  | 0.000 | 0.000 | 0.001 | 2 | 1 |  |  |
| 33 | F | 84 | Tumor_1 | intestinal | 0.000 | 0.000 | 0.001 |  |  | Positive | T2 N0 |
|  |  |  | Tumor_2 | intestinal | 0.000 | 0.000 | 0.000 |  |  |  |  |
|  |  |  | Normal |  | 0.001 | 0.000 | 0.001 | 3 | 3 |  |  |
| 34 | M | 51 | Tumor_1 | intestinal | 0.000 | 0.000 | 0.001 |  |  | Positive | T2 N0 |
|  |  |  | Tumor_2 | intestinal | 0.000 | 0.000 | 0.001 |  |  |  |  |
|  |  |  | Normal |  | 0.000 | 0.000 | 0.001 | 2 | 2 |  |  |
| 35 | M | 66 | Tumor_1 | intestinal | 0.000 | 0.000 | 0.001 |  |  | Negative | T1b N0 |
|  |  |  | Tumor_2 | intestinal | 0.001 | 0.000 | 0.001 |  |  |  |  |
|  |  |  | Normal |  | 0.001 | 0.000 | 0.001 | 0 | 0 |  |  |
| 36* | F | 75 | Tumor | intestinal | 0.000 | 0.000 | 0.001 |  |  | Positive | T1b N0 |
|  |  |  | Normal |  | 0.011 | 0.005 | 0.046 | 1 | 3 |  |  |
| 37 | M | 76 | Tumor_1 | intestinal | 0.000 | 0.000 | 0.000 |  |  | Negative | T1b N0 |
|  |  |  | Tumor_2 | intestinal | 0.000 | 0.000 | 0.000 |  |  |  |  |
|  |  |  | Normal |  | 0.001 | 0.000 | 0.001 | 0 | 0 |  |  |

**Supplementary Table 2. Clinical and pathologic factors in the training set**

| **No.** | **Sex** | **Age** | **Status** | **Lauren's type** | **IM** | **Atrophy** | **H.pylori** | **AJCC stage** |
| --- | --- | --- | --- | --- | --- | --- | --- | --- |
| 1 | M | 67 | TUMOR | Intestinal |  |  | Negative | pT1a N0 |
|  |  |  | Normal_1cm |  | 3 | 2 |  |  |
|  |  |  | Normal_3cm |  | 2 | 3 |  |  |
| 2 | M | 66 | TUMOR | Diffuse |  |  | Positive | pT1a N0 |
|  |  |  | Normal_1cm |  | 1 | 2 |  |  |
|  |  |  | Normal_3cm |  | 0 | 0 |  |  |
| 3 | M | 66 | TUMOR | Diffuse |  |  | Positive | pT1a N0 |
|  |  |  | Normal_1cm |  | 0 | 0 |  |  |
|  |  |  | Normal_3cm |  | 0 | 0 |  |  |
| 4 | F | 65 | TUMOR | Diffuse |  |  | Positive | pT1a N0 |
|  |  |  | Normal_1cm |  | 0 | 0 |  |  |
|  |  |  | Normal_3cm |  | 0 | 0 |  |  |
| 5 | F | 79 | TUMOR | Intestinal |  |  | Negative | pT2 N0 |
|  |  |  | Normal_1cm |  | 3 | 2 |  |  |
|  |  |  | Normal_3cm |  | 1 | 2 |  |  |
| 6 | M | 75 | TUMOR | Intestinal |  |  | Negative | pT1b N0 |
|  |  |  | Normal_1cm |  | 3 | 3 |  |  |
|  |  |  | Normal_3cm |  | 1 | 1 |  |  |
| 7 | M | 63 | TUMOR | Intestinal |  |  | Negative | pT1a N0 |
|  |  |  | Normal_1cm |  | 3 | 0 |  |  |
|  |  |  | Normal_3cm |  | 1 | 2 |  |  |
| 8 | M | 68 | TUMOR | Intestinal |  |  | Negative | pT1a N0 |
|  |  |  | Normal_1cm |  | 0 | 1 |  |  |
|  |  |  | Normal_3cm |  | 0 | 0 |  |  |
| 9 | M | 82 | TUMOR | Intestinal |  |  | Negative | pT1a N0 |
|  |  |  | Normal_1cm |  | 3 | 3 |  |  |
|  |  |  | Normal_3cm |  | 3 | 2 |  |  |
| 10 | F | 43 | TUMOR | Diffuse |  |  | Positive | pT1b N0 |
|  |  |  | Normal_1cm |  | 1 | 0 |  |  |
|  |  |  | Normal_3cm |  | 0 | 0 |  |  |
| 11 | M | 72 | TUMOR | Intestinal |  |  | Negative | pT1a N0 |
|  |  |  | Normal_1cm |  | 2 | 2 |  |  |
|  |  |  | Normal_3cm |  | 2 | 1 |  |  |
| 12 | F | 75 | TUMOR | Intestinal |  |  | Negative | pT1a N0 |
|  |  |  | Normal_1cm |  | 1 | 3 |  |  |
|  |  |  | Normal_3cm |  | 1 | 3 |  |  |
| 13 | M | 53 | TUMOR | Intestinal |  |  | Positive | pT1b N0 |
|  |  |  | Normal_1cm |  | 0 | 1 |  |  |
|  |  |  | Normal_3cm |  | 0 | 1 |  |  |
| 14 | M | 64 | TUMOR | Intestinal |  |  | Positive | pT1a N0 |
|  |  |  | Normal_1cm |  | 3 | 3 |  |  |
|  |  |  | Normal_3cm |  | 2 | 2 |  |  |
| 15 | M | 48 | TUMOR | Intestinal |  |  | Positive | pT2 N1 |
|  |  |  | Normal_1cm |  | 0 | 2 |  |  |
|  |  |  | Normal_3cm |  | 1 | 1 |  |  |
| 16 | M | 63 | TUMOR | Intestinal |  |  | Positive | pT1a N0 |
|  |  |  | Normal_1cm |  | 2 | 2 |  |  |
|  |  |  | Normal_3cm |  | 0 | 1 |  |  |
| 17 | M | 69 | TUMOR | Intestinal |  |  | Positive | pT1a N0 |
|  |  |  | Normal_1cm |  | 3 | 3 |  |  |
|  |  |  | Normal_3cm |  | 3 | 3 |  |  |
| 18 | F | 63 | TUMOR | Intestinal |  |  | Positive | pT1b N0 |
|  |  |  | Normal_1cm |  | 0 | 0 |  |  |
|  |  |  | Normal_3cm |  | 0 | 0 |  |  |

**Supplementary Table 3. Clinical and pathologic factors in the validation set 1**

| **No.** | **Sex** | **Age** | **Status** | **Lauren's type** | **IM** | **Atrophy** | **H.pylori** | **AJCC stage** |
| --- | --- | --- | --- | --- | --- | --- | --- | --- |
| 1 | F | 78 | TUMOR | Intestinal |  |  | Negative | pT1b N0 |
|  |  |  | Normal |  | 2 | 1 |  |  |
| 2 | M | 70 | TUMOR | Intestinal |  |  | Negative | pT1b N0 |
|  |  |  | Normal |  | 3 | 3 |  |  |
| 3 | M | 59 | TUMOR | Intestinal |  |  | Positive | pT1a N0 |
|  |  |  | Normal |  | 1 | 0 |  |  |
| 4 | M | 68 | TUMOR | Intestinal |  |  | Positive | pT1b N0 |
|  |  |  | Normal |  | 3 | 2 |  |  |
| 5 | F | 61 | TUMOR | Diffuse |  |  | Positive | pT1b N0 |
|  |  |  | Normal |  | 0 | 0 |  |  |
| 6 | M | 50 | TUMOR | Diffuse |  |  | Negative | pT1a N0 |
|  |  |  | Normal |  | 1 | 1 |  |  |
| 7 | M | 56 | TUMOR | Intestinal |  |  | Positive | pT1b N0 |
|  |  |  | Normal |  | 2 | 1 |  |  |
| 8 | M | 51 | TUMOR | Intestinal |  |  | Negative | pT1b N0 |
|  |  |  | Normal |  | 1 | 0 |  |  |
| 9 | M | 65 | TUMOR | Intestinal |  |  | Positive | pT1a N0 |
|  |  |  | Normal |  | 3 | 2 |  |  |
| 10 | F | 68 | TUMOR | Intestinal |  |  | Positive | pT1b N0 |
|  |  |  | Normal |  | 2 | 2 |  |  |
| 11 | M | 58 | TUMOR | Intestinal |  |  | Negative | pT1b N0 |
|  |  |  | Normal |  | 1 | 3 |  |  |
| 12 | F | 77 | TUMOR | Diffuse |  |  | Negative | pT1a N0 |
|  |  |  | Normal |  | 1 | 1 |  |  |
| 13 | M | 63 | TUMOR | Intestinal |  |  | Positive | pT1b N0 |
|  |  |  | Normal |  | 1 | 1 |  |  |
| 14 | F | 70 | TUMOR | Intestinal |  |  | Negative | pT1b N1 |
|  |  |  | Normal |  | 2 | 1 |  |  |
| 15 | M | 83 | TUMOR | Intestinal |  |  | Negative | pT1b N0 |
|  |  |  | Normal |  | 2 | 2 |  |  |
| 16 | M | 49 | TUMOR | Intestinal |  |  | Negative | pT1b N0 |
|  |  |  | Normal |  | 1 | 2 |  |  |
| 17 | M | 77 | TUMOR | Intestinal |  |  | Positive | pT1b N0 |
|  |  |  | Normal |  | 3 | 1 |  |  |
| 18 | M | 65 | TUMOR | Diffuse |  |  | Negative | pT1a N0 |
|  |  |  | Normal |  | 2 | 2 |  |  |
| 19 | F | 65 | TUMOR | Intestinal |  |  | Negative | pT1a N0 |
|  |  |  | Normal |  | 2 | 3 |  |  |

**Supplementary Table 4. Clinical and pathologic factors in the validation set 2**

| **No.** | **Sex** | **Age** | **Status** | **Lauren's class** | **IM** | **Atrophy** | **H.pylori** | **AJCC stage** |
| --- | --- | --- | --- | --- | --- | --- | --- | --- |
| 1 | M | 63 | Tumor_1 | intestinal |  |  | Positive | T1b N0 |
|  |  |  | Tumor_2 | intestinal |  |  |  |  |
|  |  |  | Normal |  | 1 | 1 |  |  |
| 2 | M | 48 | Tumor_1 | diffuse |  |  | Negative | T1b N0 |
|  |  |  | Tumor_2 | diffuse |  |  |  |  |
|  |  |  | Normal |  | 0 | 0 |  |  |
| 3 | F | 48 | Tumor_1 | diffuse |  |  | Positive | T1b N0 |
|  |  |  | Tumor_2 | diffuse |  |  |  |  |
|  |  |  | Normal |  | 1 | 0 |  |  |
| 4 | F | 68 | Tumor_1 | diffuse |  |  | Negative | T2 N1 |
|  |  |  | Tumor_2 | diffuse |  |  |  |  |
|  |  |  | Normal |  | 0 | 2 |  |  |
| 5 | M | 45 | Tumor_1 | intestinal |  |  | Positive | T1b N0 |
|  |  |  | Tumor_2 | intestinal |  |  |  |  |
|  |  |  | Normal |  | 0 | 0 |  |  |
| 6 | M | 64 | Tumor_1 | intestinal |  |  | Negative | T1b N1 |
|  |  |  | Tumor_2 | intestinal |  |  |  |  |
|  |  |  | Normal |  | 3 | 3 |  |  |
| 7 | F | 60 | Tumor_1 | intestinal |  |  | Positive | T1a N0 |
|  |  |  | Tumor_2 | diffuse |  |  |  |  |
|  |  |  | Normal |  | 3 | 3 |  |  |
| 8 | F | 42 | Tumor_1 | diffuse |  |  | Positive | T1b N0 |
|  |  |  | Tumor_2 | diffuse |  |  |  |  |
|  |  |  | Normal |  | 1 | 2 |  |  |
| 9 | M | 79 | Tumor_1 | intestinal |  |  | Positive | T1b N1 |
|  |  |  | Tumor_2 | intestinal |  |  |  |  |
|  |  |  | Normal |  | 3 | 3 |  |  |
| 10 | M | 66 | Tumor_1 | intestinal |  |  | Negative | T1a N0 |
|  |  |  | Tumor_2 | intestinal |  |  |  |  |
|  |  |  | Normal |  | 3 | 3 |  |  |
| 11 | M | 55 | Tumor_1 | intestinal |  |  | Negative | T1b N0 |
|  |  |  | Tumor_2 | intestinal |  |  |  |  |
|  |  |  | Normal |  | 3 | 3 |  |  |
| 12 | F | 52 | Tumor_1 | intestinal |  |  | Positive | T1b N0 |
|  |  |  | Tumor_2 | diffuse |  |  |  |  |
|  |  |  | Normal |  | 1 | 1 |  |  |
| 13 | M | 70 | Tumor_1 | intestinal |  |  | Negative | T1b N0 |
|  |  |  | Tumor_2 | intestinal |  |  |  |  |
|  |  |  | Normal |  | 3 | 3 |  |  |
| 14 | M | 63 | Tumor_1 | intestinal |  |  | Positive | T1a N0 |
|  |  |  | Tumor_2 | intestinal |  |  |  |  |
|  |  |  | Normal |  | 0 | 0 |  |  |
| 15 | F | 51 | Tumor_1 | diffuse |  |  | Positive | T1a N0 |
|  |  |  | Tumor_2 | diffuse |  |  |  |  |
|  |  |  | Normal |  | 0 | 0 |  |  |
| 16 | M | 61 | Tumor_1 | intestinal |  |  | Positive | T1b N0 |
|  |  |  | Tumor_2 | intestinal |  |  |  |  |
|  |  |  | Normal |  | 0 | 0 |  |  |
| 17 | F | 46 | Tumor_1 | diffuse |  |  | Positive | T1b N1 |
|  |  |  | Tumor_2 | diffuse |  |  |  |  |
|  |  |  | Normal |  | 0 | 0 |  |  |
| 18 | M | 60 | Tumor_1 | intestinal |  |  | Positive | T1a N0 |
|  |  |  | Tumor_2 | intestinal |  |  |  |  |
|  |  |  | Normal |  | 3 | 3 |  |  |
| 19 | M | 69 | Tumor_1 | intestinal |  |  | Positive | T1a N0 |
|  |  |  | Tumor_2 | intestinal |  |  |  |  |
|  |  |  | Normal |  | 0 | 3 |  |  |
| 20 | F | 71 | Tumor_1 | intestinal |  |  | Positive | T2 N1 |
|  |  |  | Tumor_2 | intestinal |  |  |  |  |
|  |  |  | Normal |  | 2 | 3 |  |  |
| 21 | M | 67 | Tumor_1 | intestinal |  |  | Positive | T2 N3a |
|  |  |  | Tumor_2 | intestinal |  |  |  |  |
|  |  |  | Normal |  | 3 | 3 |  |  |
| 22 | M | 71 | Tumor_1 | intestinal |  |  | Positive | T1b N0 |
|  |  |  | Tumor_2 | intestinal |  |  |  |  |
|  |  |  | Normal |  | 3 | 3 |  |  |
| 23 | M | 65 | Tumor_1 | intestinal |  |  | Positive | T1b N0 |
|  |  |  | Tumor_2 | intestinal |  |  |  |  |
|  |  |  | Normal |  | 2 | 2 |  |  |
| 24 | M | 44 | Tumor_1 | intestinal |  |  | Positive | T1a N0 |
|  |  |  | Tumor_2 | intestinal |  |  |  |  |
|  |  |  | Normal |  | 0 | 0 |  |  |
| 25 | F | 58 | Tumor_1 | intestinal |  |  | Positive | T1a N0 |
|  |  |  | Tumor_2 | intestinal |  |  |  |  |
|  |  |  | Normal |  | 3 | 3 |  |  |
| 26 | M | 61 | Tumor_1 | diffuse |  |  | Positive | T1b N0 |
|  |  |  | Tumor_2 | intestinal |  |  |  |  |
|  |  |  | Normal |  | 0 | 2 |  |  |
| 27 | M | 69 | Tumor_1 | intestinal |  |  | Negative | T1b N0 |
|  |  |  | Tumor_2 | intestinal |  |  |  |  |
|  |  |  | Normal |  | 3 | 3 |  |  |
| 28 | M | 57 | Tumor_1 | intestinal |  |  | Negative | T1a N0 |
|  |  |  | Tumor_2 | intestinal |  |  |  |  |
|  |  |  | Normal |  | 0 | 0 |  |  |
| 29 | M | 52 | Tumor_1 | intestinal |  |  | Positive | T1b N0 |
|  |  |  | Tumor_2 | intestinal |  |  |  |  |
|  |  |  | Normal |  | 1 | 0 |  |  |
| 30* | F | 40 | Tumor | diffuse |  |  | Positive | T2 N0 |
|  |  |  | Normal |  | 0 | 0 |  |  |
| 31 | F | 57 | Tumor_1 | diffuse |  |  | Negative | T1a N0 |
|  |  |  | Tumor_2 | diffuse |  |  |  |  |
|  |  |  | Normal |  | 0 | 0 |  |  |
| 32 | M | 64 | Tumor_1 | intestinal |  |  | Positive | T2 N1 |
|  |  |  | Tumor_2 | intestinal |  |  |  |  |
|  |  |  | Normal |  | 2 | 1 |  |  |
| 33 | F | 84 | Tumor_1 | intestinal |  |  | Positive | T2 N0 |
|  |  |  | Tumor_2 | intestinal |  |  |  |  |
|  |  |  | Normal |  | 3 | 3 |  |  |
| 34 | M | 51 | Tumor_1 | intestinal |  |  | Positive | T2 N0 |
|  |  |  | Tumor_2 | intestinal |  |  |  |  |
|  |  |  | Normal |  | 2 | 2 |  |  |
| 35 | M | 66 | Tumor_1 | intestinal |  |  | Negative | T1b N0 |
|  |  |  | Tumor_2 | intestinal |  |  |  |  |
|  |  |  | Normal |  | 0 | 0 |  |  |
| 36* | F | 75 | Tumor | intestinal |  |  | Positive | T1b N0 |
|  |  |  | Normal |  | 1 | 3 |  |  |
| 37 | M | 76 | Tumor_1 | intestinal |  |  | Negative | T1b N0 |
|  |  |  | Tumor_2 | intestinal |  |  |  |  |
|  |  |  | Normal |  | 0 | 0 |  |  |

* Cases with only one tumor samples (but, multiple GCs was confirmed by the record)

**Supplementary Table 5. Demographics of the cohort of healthy volunteers.**

| **No.** | **Sample No.** | **Sex** | **Age** | **IM** | **Atrophy** | **H.pylori** | **Location of biopsy** |
| --- | --- | --- | --- | --- | --- | --- | --- |
| 1 | 1 | M | 29 | 1 | 1 | Negative | Postero-GC of distal antrum |
|  | 2 |  |  | 0 | 0 |  | GC of low body |
| 2 | 3 | F | 49 | 0 | 0 | Negative | LC of mid body |
|  | 4 |  |  | 1 | 1 |  | angle |
| 3 | 5 | F | 34 | 0 | 0 | Negative | GC of mid body |
|  | 6 |  |  | 0 | 0 |  | GC of proximal antrum |
| 4 | 7 | F | 52 | 0 | 0 | Positive | GC of proximal antrum |
|  | 8 |  |  | 0 | 0 |  | GC of high body |
| 5 | 9 | M | 38 | 0 | 0 | Positive | Anterior wall of mid body |
| 6 | 10 | M | 50 | 0 | 0 | Positive | Postero-LC of proximal antrum |
| 7 | 11 | F | 45 | 0 | 0 | Positive | Anterior wall of high body |
|  | 12 |  |  | 1 | 0 |  | Postero-LC of mid antrum |
| 8 | 13 | M | 41 | 0 | 0 | Negative | GC of mid body |
|  | 14 |  |  | 0 | 0 |  | GC of proximal antrum |
| 9 | 15 | F | 45 | 0 | 0 | Negative | GC of antrum |
|  | 16 |  |  | 0 | 0 |  | GC of mid body |
| 10 | 17 | M | 44 | 0 | 0 | Positive | Antero-GC of high body |
|  | 18 |  |  | 0 | 0 |  | Anterior wall of high body |
|  | 19 |  |  | 0 | 2 |  | Posterior wall of cardia |
|  | 20 |  |  | 0 | 1 |  | LC of distal antrum |
| 11 | 21 | F | 25 | 0 | 0 | Negative | Anterior wall of antrum |
|  | 22 |  |  | 0 | 2 |  | LC of antrum |
|  | 23 |  |  | 0 | 1 |  | GC of mid body |
| 12 | 24 | F | 51 | 0 | 2 | Positive | LC of high body |
|  | 25 |  |  | 2 | 2 |  | LC of prepyloric antrum |
| 13 | 26 | M | 39 | 0 | 1 | Positive | GC of antrum |
|  | 27 |  |  | 0 | 1 |  | GC of mid body |
| 14 | 28 | F | 43 | 3 | 1 | Negative | Antero-GC of low body |
| 15 | 29 | F | 35 | 0 | 1 | Negative | pylorus |
|  | 30 |  |  | 0 | 0 |  | GC of mid body |
| 16 | 31 | F | 34 | 2 | 2 | Negative | GC of prepyloric antrum |
| 17 | 32 | F | 36 | 0 | 1 | Positive | Antero-GC of distal antrum |
| 18 | 33 | M | 49 | 0 | 1 | Positive | GC of low body |
|  | 34 |  |  | 0 | 1 |  | Postero-GC of high body |
| 19 | 35 | M | 43 | 0 | 0 | Negative | GC of low body |
|  | 36 |  |  | 0 | 0 |  | GC of mid body |
| 20 | 37 | F | 51 | 0 | 0 | Negative | Posterior wall of antrum |

**GC: greater curvature; LC: lesser curvature.**
