# Supplementary material for: Dysregulated miRNA in a cancer-prone environment: A study of gastric non-neoplastic mucosa
Source: Sci Rep. 2020 Apr 20;10:6600. doi: 10.1038/s41598-020-63230-1 (PMC7171080; doi:10.1038/s41598-020-63230-1)
Supplement: Supplementary file 4 — Supplementary Information 4. [file 41598_2020_63230_MOESM4_ESM.docx]

**Dysregulated miRNA in a cancer-prone environment: A study of gastric non-neoplastic mucosa**

Binnari Kim^1,2^, Jiryeon Jang^3^, You Jeong Heo^3^, So Young Kang^1^, Heejin Yoo^4^, Insuk Sohn^4^, Byung-Hoon Min^5,*^, and Kyoung-Mee Kim^1,2,*^

^1^Department of Pathology and Translational Genomics, Samsung Medical Center, Sungkyunkwan University School of Medicine, Seoul, Republic of Korea

^2^Center of Companion Diagnostics, Samsung Medical Center, Seoul, Republic of Korea

^3^The Samsung Advanced Institute for Health Sciences & Technology (SAIHST), Samsung Medical Center, Sungkyunkwan University School of Medicine, Seoul, Korea

^4^Statistics and Data Center, Samsung Medical Center

^5^Department of Medicine, Samsung Medical Center, Sungkyunkwan University School of Medicine, Seoul, Republic of Korea

B.K. and J.J. contributed equally to this work.

**Running title:** miRNA in the cancer-prone environment

**^*^Corresponding Authors**

Byung-Hoon Min

Department of Medicine, Samsung Medical Center, Sungkyunkwan University School of Medicine, #81, Irwon-ro, Gangnam-Gu, Seoul 06351, Korea, Phone: 82-2-3410-3409

E-mail: [Jason.min@samsung.com](mailto:Jason.min@samsung.com)

Kyoung-Mee Kim

Department of Pathology and Translational Genomics, Samsung Medical Center, Sungkyunkwan University School of Medicine, #81, Irwon-ro, Gangnam-Gu, Seoul 06351, Korea, Phone: 82-2-3410-2807, FAX: 82-2-3410-6396

E-mail: [kkmkys@skku.edu](mailto:kkmkys@skku.edu)
